# CCN1 drives asthmatic airway remodeling through amplification of TGF-β1/Smad3 signaling

**DOI:** 10.1186/s12931-026-03506-8

**Published:** 2026-01-20

**Authors:** Ying Wang, Changjuan Xu, Rong Zeng, Xiaofei Liu, Jintao Zhang, Yun Pan, Qian Qi, Chenxiao Qiao, Shuochuan Shi, Pengfei Wang, Xuemin Liu, Mingxia Gao, Tingting Gao, Liang Dong

**Affiliations:** 1 Department of Respiratory, The First Affiliated Hospital of Shandong First Medical University & Shandong Provincial Qianfoshan Hospital, Shandong Institute of Respiratory Diseases, Featured Laboratory of Respiratory Immunology and Regenerative Medicine in Universities of Shandong, Jinan Clinical Research Center for Respiratory Disease, Jinan, Shandong, 250014 China; 2 Shandong Provincial Key Medical and Health Laboratory of Translational Medicine in Microvascular Aging, The First Affiliated Hospital of Shandong First Medical University & Shandong Province Qianfoshan Hospital, Jinan, Shandong, 250014 China; 3 Beijing Ditan Hospital, Capital Medical University, Beijing, China

**Keywords:** Asthma, Airway remodeling, CCN1, Epithelial-Mesenchymal transition, Extracellular matrix, TGF-β1

## Abstract

**Background:**

Airway remodeling contributes significantly to chronic airflow limitation in asthma, but effective therapies are lacking. The matricellular protein CCN1 is implicated in fibrosis across multiple organ systems; however, its specific contribution to the pathogenesis of asthmatic airway remodeling has not been clearly delineated. This study was therefore designed to elucidate the functional role and regulatory mechanisms of CCN1 in this disease context.

**Methods:**

To establish clinical relevance, we first compared CCN1 expression levels in bronchial biopsies and serum samples obtained from asthmatic patients versus those from healthy controls. To investigate its functional role in vivo, we utilized a murine model of ovalbumin (OVA)-induced chronic asthma, where the effects of CCN1 were interrogated using both shRNA-mediated knockdown and intranasal administration of recombinant protein. In parallel in vitro studies, we exposed human bronchial epithelial cells (BEAS-2B) to TGF-β1 and employed siRNA-mediated silencing or plasmid-driven overexpression to assess the impact of CCN1 on epithelial-mesenchymal transition (EMT) and cellular migration. The signaling mechanism was explored by analyzing Smad3 phosphorylation and its subcellular localization.

**Results:**

We observed a significant upregulation of CCN1 within both the airway mucosa and serum of asthmatic individuals, correlating with remodeling features. In the mouse model, CCN1 expression was similarly upregulated. Knockdown of CCN1 attenuated airway hyperresponsiveness, remodeling, and EMT, whereas exogenous CCN1 administration exacerbated these pathologies. In vitro, TGF-β1 was identified as a potent inducer of CCN1 expression in bronchial epithelial cells. Mechanistically, CCN1 was both necessary and sufficient for TGF-β1-driven EMT and pro-fibrotic responses. Notably, we unveiled a novel positive feedback loop initiated by TGF-β1-induced CCN1. This CCN1 induction, in turn, amplified canonical TGF-β1 signaling by promoting the phosphorylation and subsequent nuclear translocation of Smad3.

**Conclusions:**

Our study identifies CCN1 as an important mediator of asthmatic airway remodeling. We demonstrate that CCN1 is induced by TGF-β1 and, in turn, enhances TGF-β1/Smad3 signaling, creating a positive feedback mechanism that promotes pro-fibrotic and EMT-like responses in bronchial epithelial cells. Collectively, these results highlight the CCN1 axis as a promising therapeutic target for mitigating airway remodeling in asthma.

**Supplementary Information:**

The online version contains supplementary material available at 10.1186/s12931-026-03506-8.

## Background

Asthma represents a major global health burden, affecting hundreds of millions of individuals and imposing substantial costs on healthcare systems worldwide [1]. Pathophysiologically, the disease is characterized by two intertwined features: chronic airway inflammation and a series of structural changes collectively known as airway remodeling [2]. While once considered a late-stage consequence of chronic inflammation, compelling evidence now indicates that airway remodeling begins early in life—detectable even in preschool children—and persists throughout the disease course [3, 4]. Despite the availability of effective anti-inflammatory and bronchodilator therapies, current management strategies do not prevent or reverse airway remodeling, a hallmark of long-standing asthma that contributes to progressive lung function decline and persistent symptoms [5].

A key hallmark of these structural alterations is a profound dysregulation of the extracellular matrix (ECM), which manifests as its excessive deposition, altered composition, and increased stiffness within the airway wall [6]. This intricate network of secreted proteins and polysaccharides provides not only the physical scaffolding for the airway but also a rich source of biochemical cues that direct cell behavior [7, 8]. Therefore, identifying the key molecular players that orchestrate ECM dysregulation is critical for developing new strategies to prevent or reverse airway remodeling in asthma.

The matricellular protein CCN1, formerly known as cysteine-rich angiogenic inducer 61 (CYR61), serves as the prototypical member of the six-protein CCN family (CCN1-6). Rather than providing primary structural support within the ECM, these proteins act as critical signaling hubs, modulating the dialogue between cells and their microenvironment [9]. Notably, the synthesis of CCN1 is potently induced by a range of stimuli, including growth factors, key pro-inflammatory cytokines such as TNF-α and TGF-β1, and biophysical cues like mechanical stress [10–12]. This inducibility places CCN1 at the center of many dynamic biological processes [13, 14]. However, the sustained, chronic expression of CCN1 is a hallmark of pathological remodeling and fibrosis. In fibrotic contexts, this sustained and aberrant CCN1 expression initiates a pathological cascade that includes persistent fibroblast activation, differentiation into myofibroblasts, and culminates in excessive ECM deposition [15]. This pathogenic role has been extensively documented in a range of fibrotic disorders, including pulmonary fibrosis [9, 16], liver fibrosis [17, 18], kidney fibrosis, and scleroderma, underscoring its significance as a pro-fibrotic mediator. Despite its well-documented role as a pro-fibrotic mediator in various chronic diseases, the involvement of CCN1 in asthma-related airway remodeling remains largely unexplored. Therefore, the present study was designed to investigate the function and underlying mechanisms of CCN1 in this context, aiming to establish its significance in the pathogenesis of asthma.

## Materials and methods

### Human subjects and sample collection

All procedures involving human participants were reviewed and approved by the Ethics Committee of The First Affiliated Hospital of Shandong First Medical University. We enrolled two distinct cohorts recruited from this institution: one group comprised 25 patients with a confirmed diagnosis of asthma according to current GINA criteria, and the second was a corresponding group of 25 age-and sex-matched healthy volunteers. For serum preparation, peripheral blood samples were drawn and permitted to clot at ambient temperature for 30 min. The clotted samples were then subjected to centrifugation at 3,000 rpm for 10 min. The resulting supernatant serum was carefully aspirated, dispensed into aliquots, and cryopreserved at −80 °C until required for analysis. Additionally, bronchial mucosal biopsies were obtained via bronchoscopy from selected participants (an asthma patient and a healthy control). Control tissue was sourced from a macroscopically normal airway region in an individual who underwent bronchoscopy but was ultimately found to have no pathological abnormalities. The clinical characteristics of the subjects are detailed in Additional file 1: Table S1.

### Animal model and experimental treatments

To establish a chronic allergic airway inflammation phenotype, we utilized a well-characterized OVA-induced asthma model. Female C57BL/6 mice (6–8 weeks of age, ~ 20 g) were procured from Beijing Vital River Laboratory Animal Technology Co., Ltd. (Beijing, China) and were housed under specific-pathogen-free (SPF) conditions. The sensitization phase was initiated via a series of intraperitoneal (i.p.) injections on days 0, 7, and 14, with each injection containing 20 µg of OVA (Sigma-Aldrich) emulsified in a 2 mg aluminum hydroxide adjuvant. Subsequently, from day 21 to 57, mice underwent airway challenges every other day with a 3% OVA aerosol. For the loss-of-function study, mice were assigned to four groups (*n* = 5 per group): Lv2-shNC, Lv2-shCCN1, OVA + Lv2-shNC, and OVA + Lv2-shCCN1. The OVA groups were subjected to the asthma protocol, while the non-OVA groups received saline. All groups received intravenous injections of either CCN1-targeting shRNA lentivirus (Lv2-shCCN1) or a non-targeting control (Lv2-shNC) (GenePharma, Shanghai, China) on days 21, 35, and 49. In the complementary gain-of-function study, mice were allocated to three groups (*n* = 5 each): Control, OVA, and OVA + rmCCN1. The OVA and OVA + rmCCN1 groups were subjected to the asthma protocol. On day 21 and weekly thereafter, the OVA + rmCCN1 group received intranasal recombinant mouse CCN1 (0.5 µg; MedchemExpress, HY-P704010), while the OVA and Control groups received PBS vehicle. At 24 h following the final OVA challenge, we assessed airway hyperresponsiveness (AHR) in response to escalating doses of methacholine using a FlexiVent system. Mice were then euthanized for tissue collection.

### Enzyme-linked immunosorbent assay (ELISA)

We quantified CCN1 protein levels in serum samples employing a commercial ELISA kit (Cat# KYY-13673H1, Shandong Keyan Yun Biotechnology), strictly adhering to the manufacturer’s instructions.

### Cell culture and treatments

BEAS-2B cells were obtained from Shanghai Fuheng Biotechnology Co., Ltd. (Xian, China) and maintained in DMEM/F12 medium supplemented with 10% FBS and 1% penicillin-streptomycin. For all experiments, cells were serum-starved (0.5% FBS) for 12 h prior to treatment. Recombinant human TGF-β1 (PRP100190, Abbkine, Wuhan, China) was used for dose-response (0–20 ng/ml for 24 h) and time-course (10 ng/ml for 0–48 h) experiments.To investigate the function and mechanism of CCN1, we transfected BEAS-2B cells with small interfering RNA (siRNA) negative control or CCN1 siRNA (GenePharma, Shanghai, China), control vector or overexpression CCN1 vector (GenePharma, Shanghai, China) using EndoFectin™ Max Transfection Reagent (GeneCopoeia, Guangzhou, China) according to the manufacturer’s instruction. Four to six hours after transfection, the culture medium was replaced with fresh medium containing TGF-β1 (10 ng/ml), and cells were cultured for a further 48 h.The sequences for the siRNAs used are provided in Additional file 1: Table S2.

### Western blotting

To prepare protein lysates, cultured cells or homogenized lung tissues were lysed on ice using ice-cold radio immunoprecipitation assay lysis buffer (RIPA) fortified with a commercial protease and phosphatase inhibitor cocktail. Protein concentration of the resulting lysates was determined using a bicinchoninic acid (BCA) protein assay kit. For electrophoresis, an equal mass of protein (20–30 µg) per lane was separated on SDS-polyacrylamide gels and subsequently electroblotted onto polyvinylidene difluoride (PVDF) membranes. To minimize non-specific antibody binding, membranes were incubated in a blocking buffer (5% non-fat milk in Tris-buffered saline with 0.1% Tween-20) for 1 h at room temperature. For immunoblotting of phosphoproteins, the blocking solution was replaced with 5% bovine serum albumin (BSA) in the same buffer. Membranes were then probed overnight at 4 °C with the relevant primary antibodies listed below: anti-CCN1 (1:1,000; Cell Signaling Technology, cat.no. 14479), anti-Collagen I (1:1,000; Abcam, cat.no.ab260043), anti-alpha-smooth muscle actin (α-SMA) (1:1,000; HUABIO, cat.no.ET1607-53), anti-Fibronectin (1:1,000; HUABIO, cat.no.ET1702-25), anti-E-cadherin (1:1,000; Cell Signaling Technology, cat.no.3195), anti-N-cadherin (1:1,000; Cell Signaling Technology, cat.no.13116), anti-Vimentin (1:1,000; Cell Signaling Technology, cat.no.5741), anti-Smad3 (1:1,000; HUABIO, cat.no.ET1607-41), and anti-phospho-Smad3 (1:1,000; HUABIO, cat.no. ET1609-41). Following incubation with the appropriate HRP-conjugated secondary antibodies, immunoreactive bands were detected using an enhanced chemiluminescence (ECL) substrate. The resulting signals were captured, and band intensity was quantified using ImageJ software.

### Quantitative real-time PCR (RT-qPCR)

Total RNA was isolated using the Super FastPure Cell RNA Isolation Kit (RC102-01, Vazyme, Nanjing, China) and reverse-transcribed into cDNA. Gene expression was quantified by RT-qPCR on a Bio-Rad CFX96 platform using a SYBR Green-based master mix (Vazyme, Nanjing, China). We calculated relative mRNA abundance using the 2^^−ΔΔCt^ method, normalizing expression levels to the internal reference gene, GAPDH. Each reaction was run in technical triplicate. The specificity of the amplification product was verified through a final melting curve analysis. All primer sequences are provided in Additional file 1: Table S3.

### Histology and immunostaining

Paraffin-embedded lung sections were deparaffinized, rehydrated, and subjected to heat-induced antigen retrieval in citrate buffer (pH 6.0). For immunohistochemistry (IHC), endogenous peroxidase activity was subsequently quenched, and sections were blocked prior to being probed overnight at 4 °C with primary antibodies. The antibodies used were: anti-CCN1 (1:200; HUABIO, cat.no.HA723070), anti-E-cadherin (1:1,000; Cell Signaling Technology, cat.no.3195), anti-N-cadherin (1:1,000; Cell Signaling Technology, cat.no.13116), anti-alpha-smooth muscle actin (α-SMA) (1:1,000; HUABIO, cat.no.ET1607-53), anti-Fibronectin (1:1,000; HUABIO, cat.no.ET1702-25), anti-Collagen I (1:200; BOSTER, BA0325), and anti-Smad3 (1:1,000; HUABIO, cat.no.ET1607-41). Signals were developed using an HRP-conjugated secondary antibody and a DAB substrate kit, followed by hematoxylin counterstaining. For immunofluorescence (IF), following primary antibody incubation, lung sections were probed with the appropriate Alexa Fluor-conjugated secondary antibodies and counterstained with DAPI to visualize nuclei. A similar IF protocol was applied to cultured BEAS-2B cells, which were first fixed and permeabilized before being stained for Smad3. All samples were imaged on a light or confocal microscope. Staining intensity was quantified as the integrated optical density (IOD) or mean fluorescence intensity (MFI) from at least five random fields per sample using ImageJ.

### Cell migration assay

We evaluated cellular migratory capacity using Transwell inserts featuring an 8-µm pore membrane. To initiate the assay, a cell suspension of 1 × 10⁵ BEAS-2B cells in serum-free medium was loaded into the apical (upper) chamber. A chemoattractant gradient was then generated by filling the basolateral (lower) chamber with medium containing 10% FBS. After a 24-hour incubation, non-migratory cells adhering to the upper surface of the membrane were gently removed with a cotton swab. For quantification, cells that had traversed the membrane to the lower surface were fixed with 4% paraformaldehyde, stained with a 0.5% crystal violet solution, and imaged. The number of migrated cells was then enumerated by counting five independent microscopic fields per insert to determine the average migratory response.

### Nuclear and cytoplasmic fractionation

To separate nuclear and cytoplasmic components, we performed cell fractionation using a commercial subcellular fractionation kit, with strict adherence to the manufacturer’s specified protocol. To confirm the purity and integrity of these subcellular lysates, we performed validation by immunoblotting. The successful separation was verified by probing for the presence of the cytoplasmic marker GAPDH and the nuclear-specific marker Lamin B1. A clean fractionation was confirmed by the exclusive detection of Lamin B1 in the nuclear fraction and GAPDH in the cytoplasmic fraction.

### Statistical analysis

All quantitative data are expressed as the mean ± standard deviation (SD) derived from a minimum of three independent experiments. All statistical computations were performed using GraphPad Prism (Version 10). To assess significant differences between two experimental groups, an unpaired, two-tailed Student’s t-test or a non-parametric Mann-Whitney U test was employed based on data distribution. For analyses involving three or more experimental groups, a one-way or two-way analysis of variance (ANOVA) was performed, which was followed by an appropriate multiple comparisons test to identify specific inter-group differences. Across all statistical tests, a *P*-value of < 0.05 was established as the criterion for statistical significance.

## Results

### CCN1 is upregulated in human asthma and drives key features of airway remodeling in a chronic murine model

**Fig. 1:**
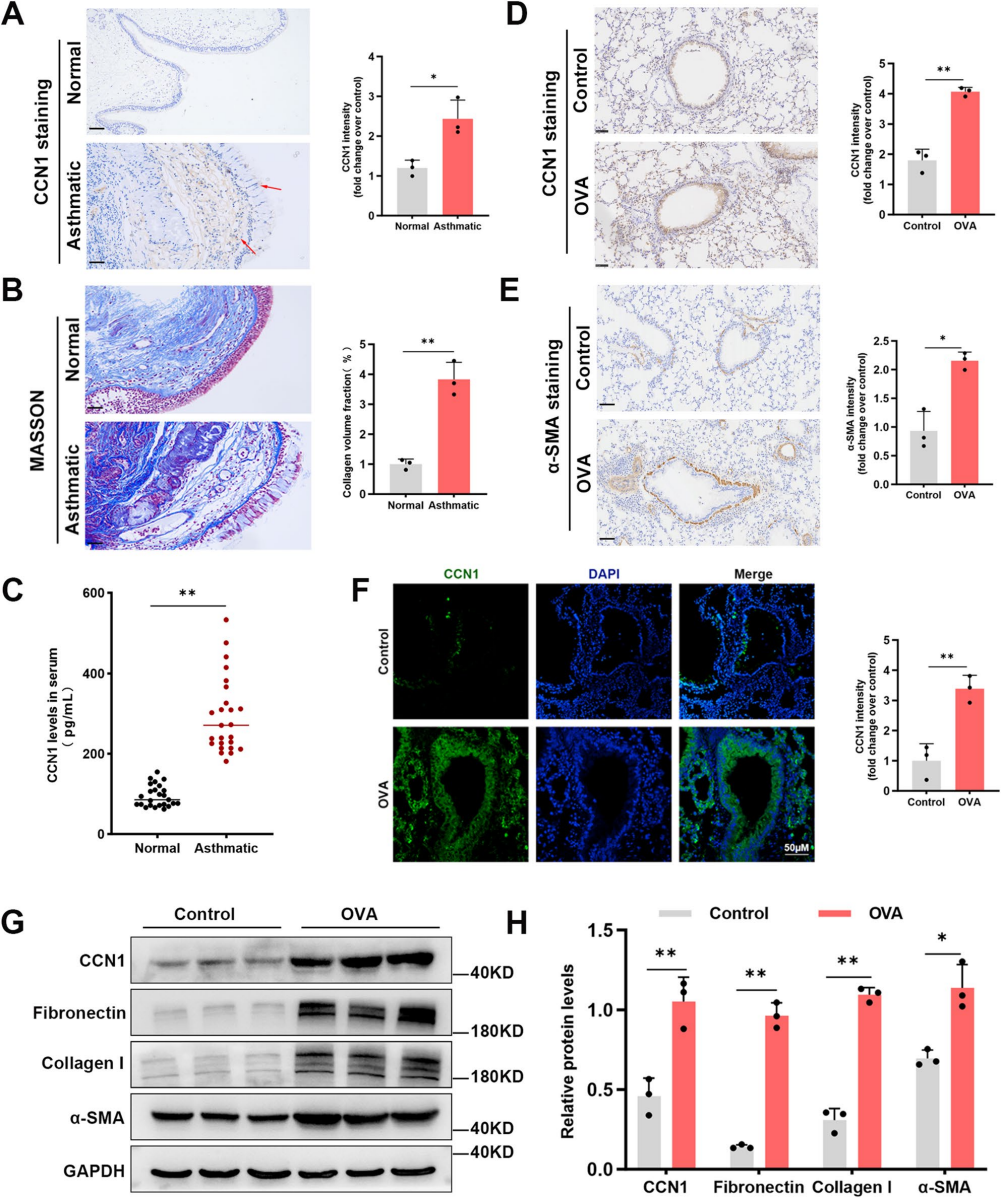
CCN1 expression is upregulated in asthmatic patients and a murine model of chronic allergic airway inflammation. (**A**) Representative IHC staining and quantification of CCN1 in bronchial biopsies from asthmatic patients and healthy controls. Arrows indicate enhanced CCN1 expression. (**B**) Representative Masson’s trichrome staining showing collagen deposition (blue) and its quantification in adjacent sections from the same patient cohort. (**C**) Serum CCN1 levels in asthmatic patients and healthy controls, as measured by ELISA. Each dot represents an individual subject. (**D**,** E**) Immunohistochemical staining and corresponding quantification assessing CCN1 (**D**) and α-SMA (**E**) expression in lung sections from mice chronically challenged with either OVA or saline. (**F**) Immunofluorescence microscopy was used to visualize CCN1 expression (green) within the lung tissue of OVA-challenged mice compared to saline-treated controls. Cell nuclei were identified with a DAPI counterstain (blue). (**G**,** H**) The expression of CCN1 and key pro-remodeling proteins (Fibronectin, Collagen I, α-SMA) in whole-lung homogenates was quantified by immunoblotting (**G**) with corresponding densitometry (**H**). GAPDH served as the internal loading control. Scale bars = 50 μm. All quantitative data are expressed as mean ± SD. Statistical significance between two groups was assessed using an unpaired, two-tailed Student’s t-test. ^*^P < 0.05; ^**^P < 0.01

To assess the potential involvement of CCN1 in asthma, we first analyzed its expression in bronchial biopsies from patients and healthy controls. IHC staining revealed significantly higher CCN1 expression in the airway mucosa of asthmatic patients, predominantly within the bronchial epithelium and submucosal layers as indicated by arrows (Fig. 1A). As expected, these same patients exhibited classic features of airway remodeling, including subepithelial fibrosis and increased collagen deposition, confirmed by Masson’s trichrome staining on adjacent sections (Fig. 1B). We next asked whether this local upregulation was reflected systemically. Indeed, an ELISA showed that serum CCN1 concentrations were also significantly elevated in patients with asthma compared to healthy individuals (Fig. 1C).

Having established this link in human asthma, we sought to determine if it was recapitulated in a murine model of chronic allergic airway disease. In mice sensitized and challenged with OVA, IHC analysis showed a marked increase in CCN1 expression within the bronchial epithelium, a finding confirmed by immunofluorescence (Fig. 1D and F). This was accompanied by significant peribronchial smooth muscle thickening, a hallmark of airway remodeling confirmed by specific α-SMA staining localized to the peribronchial smooth muscle layer (Fig. 1E). To corroborate these histological findings at the molecular level, we performed Western blot analysis of whole-lung homogenates. This analysis not only confirmed the significant increase in CCN1 but also showed a concurrent upregulation of key remodeling-associated proteins, including Fibronectin, Collagen I, and α-SMA, in mice subjected to the OVA challenge (Fig. 1G and H).

Collectively, these data from both human patients and a corresponding animal model establish a strong association between elevated CCN1 expression and the pathological hallmarks of airway remodeling.

### CCN1 knockdown attenuates airway hyperresponsiveness and remodeling in a chronic asthma model

**Fig. 2:**
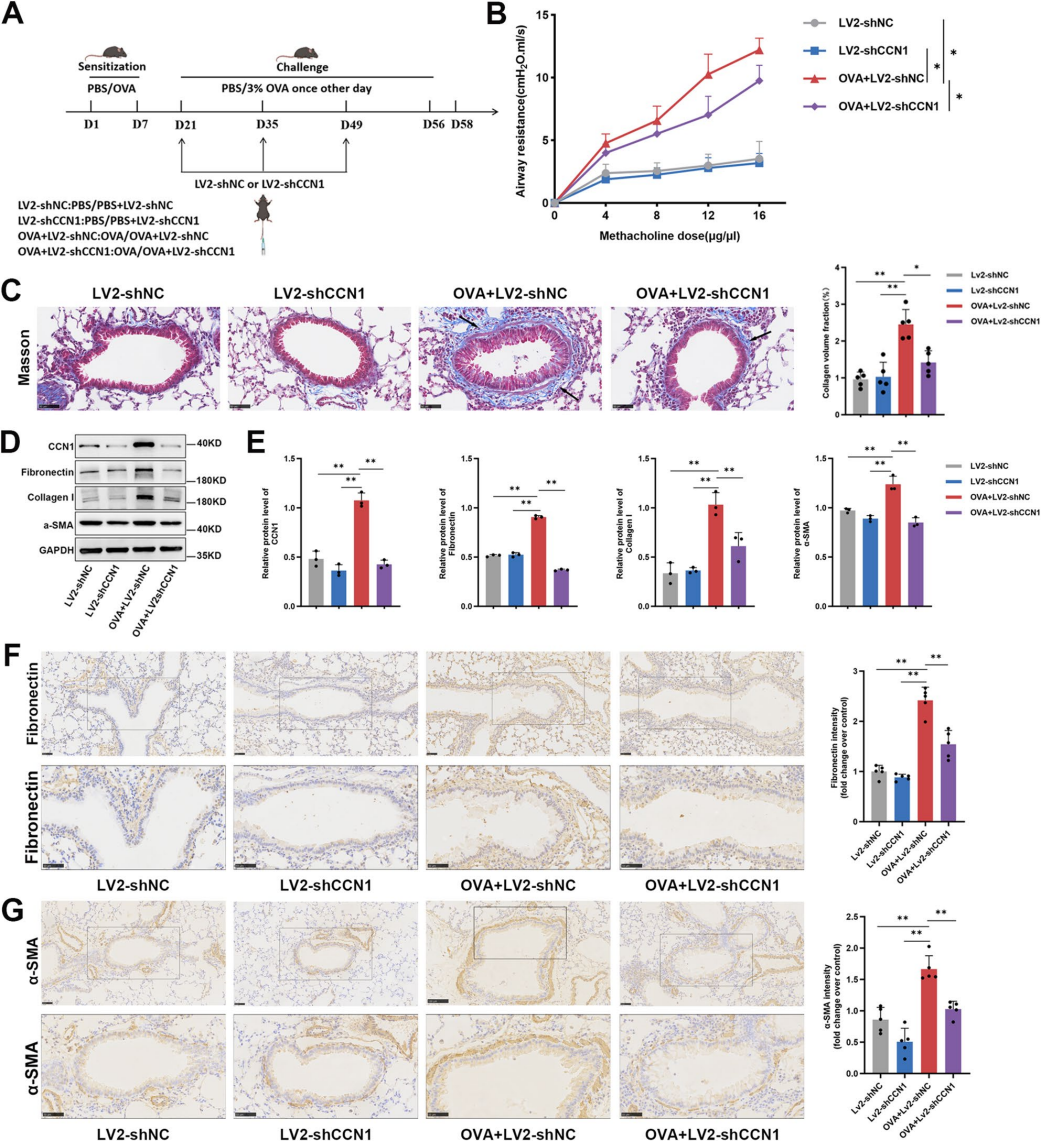
Lentiviral-mediated knockdown of CCN1 alleviates AHR and remodeling in a murine model of chronic asthma. (**A**) A schematic illustrating the experimental timeline for the chronic OVA challenge model combined with therapeutic delivery of lentivirus carrying either LV2-shCCN1 or LV2-shNC. (**B**) Airway hyperresponsiveness was evaluated by measuring airway resistance in response to escalating doses of aerosolized methacholine across the four experimental groups (*n* = 5 per group). (**C**) Subepithelial fibrosis was visualized by Masson’s trichrome staining. Representative images and corresponding quantification of the peribronchial collagen area (blue) in lung sections are shown. Arrows highlight areas of enhanced collagen deposition. (**D**,** E**) The expression of CCN1 and key pro-fibrotic proteins (Fibronectin, Collagen I, α-SMA) was quantified in whole-lung homogenates via immunoblotting (**D**) with corresponding densitometry (**E**). (**F**,** G**) Immunohistochemical staining was used to assess the protein expression of Fibronectin (**F**) and α-SMA (**G**) in lung sections. For each marker, the bottom panels display higher-magnification views of the boxed regions shown in the top panels For all blots, GAPDH served as the internal loading control. Scale bars = 50 μm. All quantitative data are expressed as mean ± SD. Statistical comparisons for the AHR data (B) were performed using a two-way ANOVA. All other comparisons were made using a one-way ANOVA with Tukey’s multiple comparisons test. ^*^P < 0.05 and ^**^P < 0.01 were considered statistically significant vs. the relevant control group

Given the strong correlation between CCN1 expression and airway remodeling, we next sought to determine whether CCN1 plays a direct functional role in asthma pathogenesis. To this end, we employed a loss-of-function approach, using a lentiviral-mediated shRNA to knock down CCN1 in our chronic OVA-challenge model (Fig. 2A). This intervention successfully suppressed the OVA-induced upregulation of CCN1 within the airway epithelium (Additional file 1: Figure S1A-B).

First, we assessed the impact of CCN1 knockdown on AHR, a key functional deficit in asthma. As expected, mice challenged with OVA (OVA + Lv-shNC) displayed a robust AHR to increasing doses of methacholine. Notably, this hyperresponsiveness was significantly blunted in mice where CCN1 was knocked down (OVA + Lv-shCCN1), bringing airway resistance to levels much closer to those of non-challenged controls (Fig. 2B).

This functional recovery was mirrored by structural improvements at the histological level. Masson’s trichrome staining revealed that the extensive peribronchial collagen deposition and subepithelial fibrosis seen in the OVA + Lv-shNC group (highlighted by arrows) were substantially reduced in mice from the OVA + Lv-shCCN1 group, suggesting that CCN1 is critical for this fibrotic process (Fig. 2C). To corroborate these histological observations at the molecular level, we analyzed lung homogenates via Western blotting. This analysis not only confirmed the efficacy of our knockdown strategy—evidenced by significantly reduced CCN1 protein levels—but also demonstrated that the increased expression of key remodeling markers (Fibronectin, Collagen I, and α-SMA) observed in the OVA + Lv-shNC group was largely reversed in mice receiving the CCN1-targeting shRNA (Fig. 2D and E).

Finally, we verified the spatial distribution of these markers using immunohistochemistry (IHC). Consistent with the Western blot results, the intense peribronchial staining for both Fibronectin and α-SMA was visibly diminished upon CCN1 knockdown (Fig. 2F and G). Collectively, these data indicate that CCN1 is a critical mediator of both airway hyperresponsiveness and structural remodeling in our model of chronic allergic asthma.

### CCN1 drives an EMT-like program in the airway epithelium of asthmatic mice

EMT, a process wherein epithelial cells adopt mesenchymal traits, is now recognized as a significant driver of airway remodeling [6]. We therefore hypothesized that CCN1 might exert its pro-remodeling effects by inducing EMT in the airway epithelium. To investigate this, we analyzed key EMT markers in the lung tissues from our chronic asthma model.

**Fig. 3:**
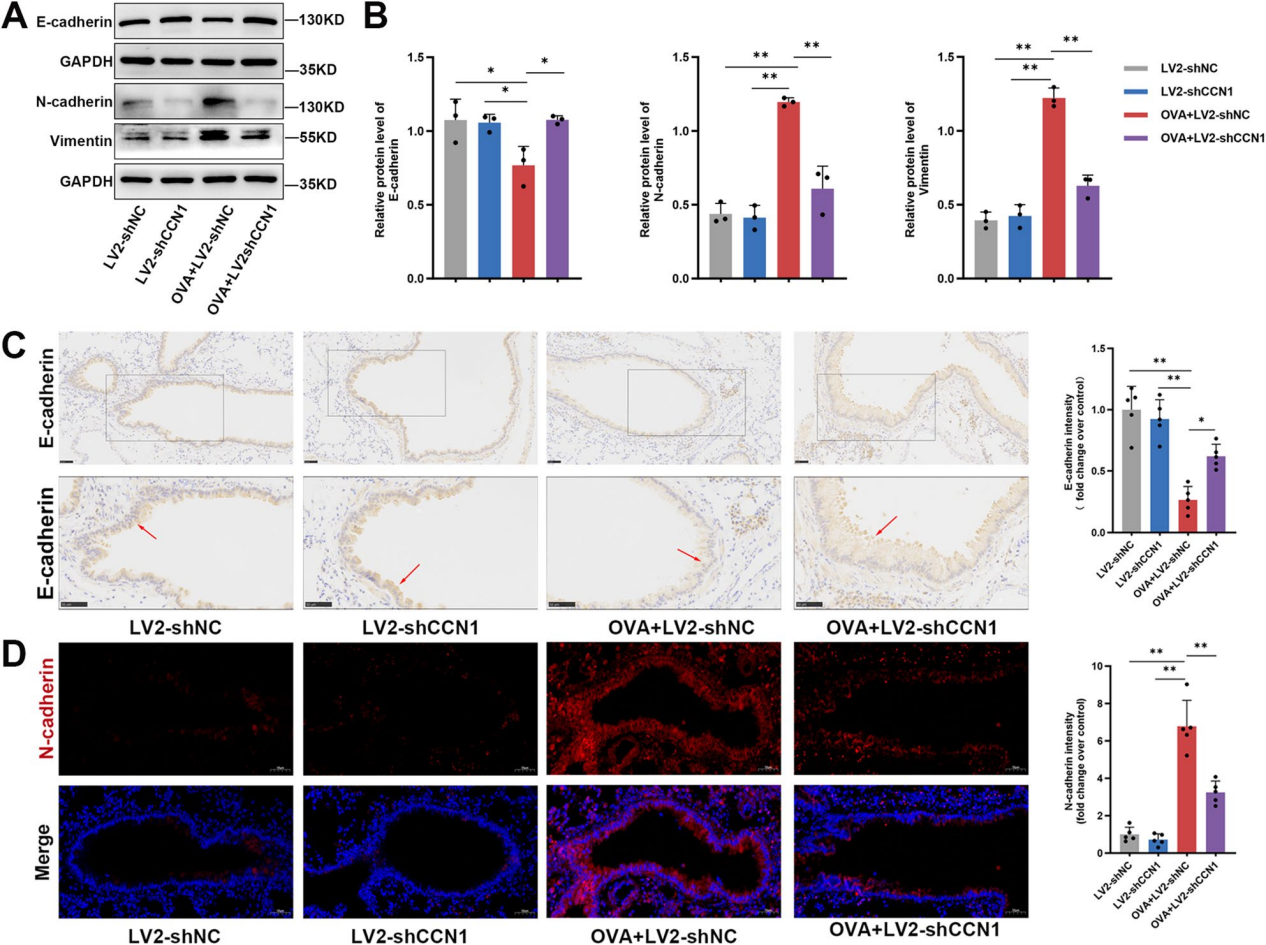
Targeted knockdown of CCN1 reverses key features of EMT in the airways of asthmatic mice. (**A**, **B**) Immunoblot analysis (**A**) and associated densitometry (**B**) quantifying the expression of the epithelial marker E-cadherin alongside the mesenchymal markers N-cadherin and Vimentin in whole-lung homogenates. GAPDH served as the internal loading control for all blots. (**C**) Immunohistochemical analysis was performed to assess E-cadherin expression in lung sections. The bottom panels provide magnified insets of the boxed regions from the top panels, allowing for detailed visualization of epithelial cell-cell junction integrity. Arrows indicate regions of disrupted or reduced E-cadherin staining at the cell junctions. (**D**) Immunofluorescence microscopy was used to visualize N-cadherin expression (red) specifically within the airway epithelium. Cell nuclei were identified with a DAPI counterstain (blue) Scale bars represent 50 μm. All quantitative data are expressed as the mean ± SD, with *n* = 3–5 mice per experimental group. Group comparisons were performed using one-way ANOVA with Tukey’s multiple comparisons test. ^*^P < 0.05 and ^**^P < 0.01 were considered statistically significant vs. the relevant control group

Western blot analysis of lung homogenates revealed a classic EMT signature in OVA-challenged mice. Lungs from the OVA + Lv-shNC group displayed a classic EMT signature, characterized by a pronounced loss of the epithelial marker E-cadherin alongside a concomitant upregulation of the mesenchymal markers N-cadherin and Vimentin (Fig. 3A and B). Importantly, this EMT-like molecular profile was largely reversed by CCN1 knockdown. In the OVA + Lv-shCCN1 group, E-cadherin expression was substantially restored, while the induction of N-cadherin and Vimentin was significantly attenuated (Fig. 3A and B).

To visualize these changes within the tissue architecture, we performed IHC and IF staining. In the OVA + Lv-shNC group, IHC staining showed that E-cadherin expression was not only diminished but also displayed discontinuous localization at cell-cell junctions in the airway epithelium—a hallmark of EMT. This loss of epithelial integrity was largely prevented in mice from the OVA + Lv-shCCN1 group, where strong and continuous E-cadherin staining was maintained at cell junctions (indicated by arrows, Fig. 3C). Conversely, IF staining revealed a pronounced upregulation of N-cadherin in the airway epithelium of OVA-challenged mice, an effect that was markedly suppressed following CCN1 knockdown (Fig. 3D). Taken together, these molecular and histological data strongly suggest that CCN1 promotes airway remodeling, at least in part, by driving an EMT-like program in the airway epithelium.

### Exogenous CCN1 exacerbates airway remodeling and EMT in asthmatic mice

**Fig. 4:**
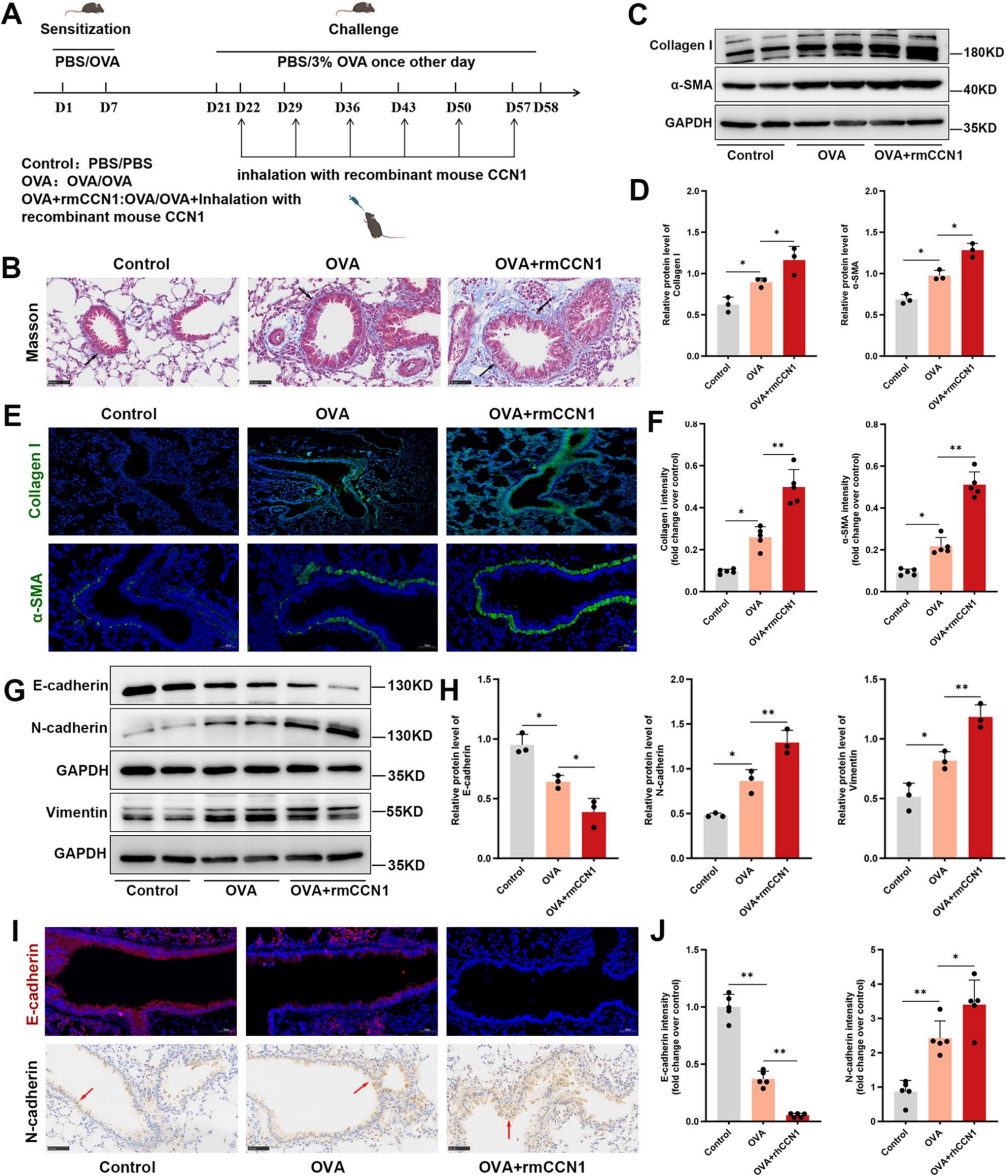
Exogenous CCN1 exacerbates OVA-induced airway remodeling and EMT. (**A**) A schematic diagram illustrating the experimental design for the chronic OVA challenge model combined with intranasal administration of rmCCN1. (**B**) Representative Masson’s trichrome staining showing peribronchial collagen deposition (blue) in lung sections from Control, OVA, and OVA + rmCCN1 treated mice. Arrows indicate areas of collagen deposition. (**C**, **D**) Immunoblot analysis (**C**) and corresponding densitometry (**D**) quantifying the expression of pro-fibrotic markers, Collagen I and α-SMA, in whole-lung homogenates. GAPDH served as the internal loading control. (**E**, **F**) Representative IF images (**E**) and quantification (**F**) for Collagen I (green, upper panel) and α-SMA (green, lower panel) in lung sections. Nuclei were counterstained with DAPI (blue). (**G**, **H**) Immunoblot analysis (**G**) and corresponding densitometry (**H**) quantifying the expression of the epithelial marker E-cadherin and the mesenchymal markers N-cadherin and Vimentin. Respective GAPDH bands are shown as internal loading controls for each gel. (**I**, **J**) Representative IF images for E-cadherin (red, upper panel) and IHC for N-cadherin (brown, lower panel, indicated by red arrows) in lung sections (**I**), with their corresponding quantification (**J**). Scale bars represent 50 μm. All quantitative data are shown as the mean ± SD, with *n* = 3–5 mice per group. Statistical comparisons were performed via one-way ANOVA with Tukey’s multiple comparisons test. ^*^P < 0.05 and ^**^P < 0.01 were considered significant vs. the relevant control group

Our loss-of-function experiments indicated that CCN1 is necessary for OVA-induced airway remodeling. To complement these findings, we performed a gain-of-function experiment to determine if exogenous CCN1 was sufficient to exacerbate this pathology. Recombinant mouse CCN1 (rmCCN1) was administered intranasally to mice during the OVA challenge protocol (Fig. 4A).

The administration of rmCCN1 markedly amplified the structural changes in the airways. Masson’s trichrome staining revealed that subepithelial collagen deposition was significantly exacerbated in the OVA + rmCCN1 group compared to the OVA-challenged mice, with arrows highlighting the intensified fibrosis (Fig. 4B). This intensified fibrotic response was corroborated at the molecular level, where analyses confirmed that Collagen I and α-SMA expression were further augmented in the peribronchial regions of mice receiving rmCCN1 (Fig. 4C and D). Furthermore, immunofluorescence staining visually confirmed the enhanced deposition of these remodeling markers (Fig. 4E and F).

We next examined if this heightened remodeling correlated with a more pronounced EMT program. Indeed, Western blot analysis showed that the OVA-induced loss of E-cadherin was more severe in the OVA + rmCCN1 group. Conversely, the expression of the mesenchymal markers N-cadherin and Vimentin was further augmented by rmCCN1 administration (Fig. 4G and H). Tissue staining provided visual confirmation of these molecular changes, with immunofluorescence for E-cadherin and immunohistochemistry for N-cadherin revealing a more profound loss of epithelial integrity and a stronger induction of the mesenchymal phenotype in the airways of the OVA + rmCCN1 group (Fig. 4I and J). Together, these gain-of-function data demonstrate that elevated CCN1 levels are sufficient to aggravate both airway remodeling and the underlying EMT program in the context of allergic airway inflammation.

### TGF-β1 induces CCN1 expression and promotes an EMT phenotype in human bronchial epithelial cells

Having observed the association between CCN1 and airway remodeling in our in vivo model, we next sought to delineate the upstream regulatory mechanisms governing CCN1 expression. Given the central role of TGF-β1 in asthmatic fibrosis, we utilized BEAS-2B to investigate the regulation of CCN1 by TGF-β1 signaling.

**Fig. 5:**
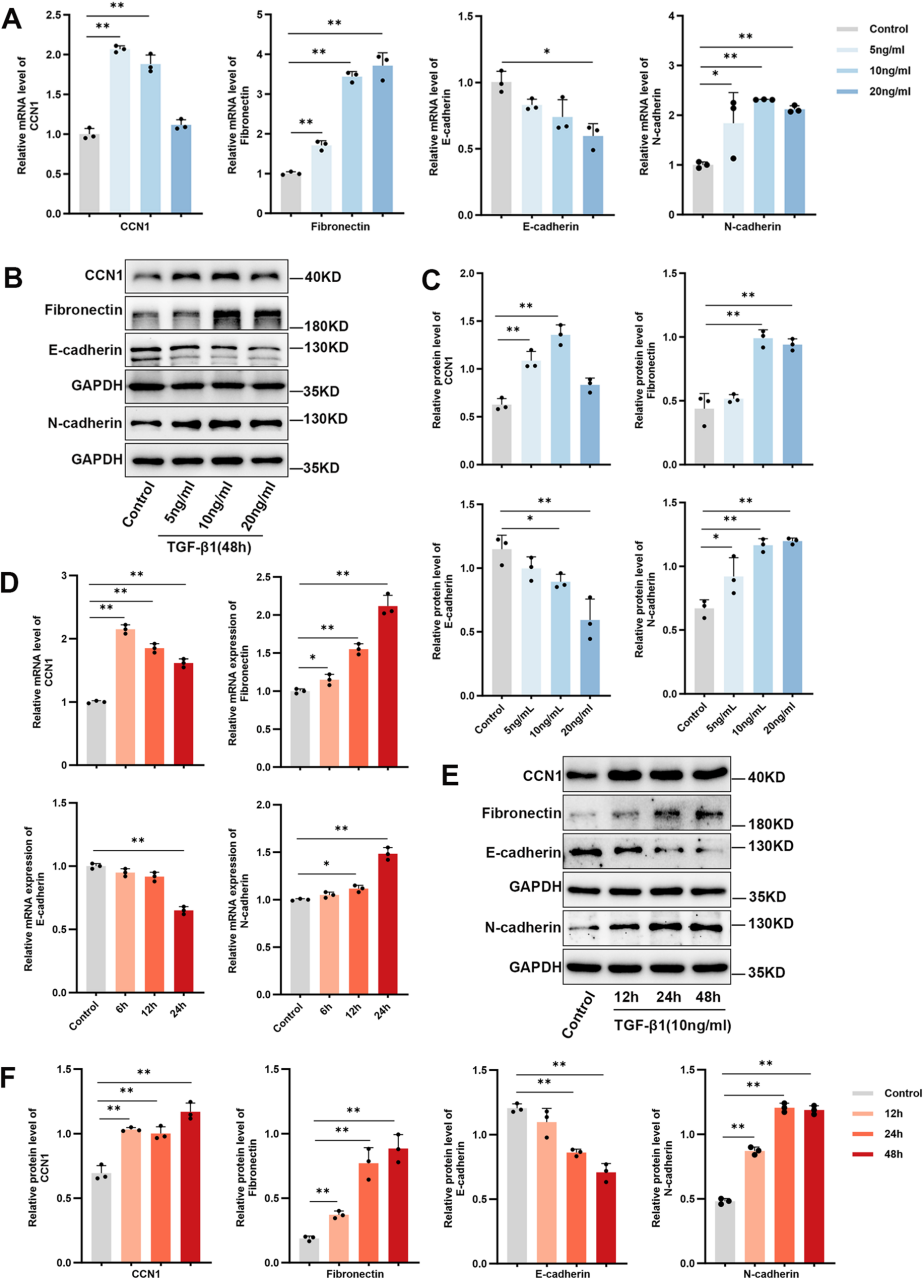
TGF-β1 modulates CCN1 expression and induces an EMT-like phenotype in BEAS-2B cells (**A**) Effect of escalating TGF-β1 concentrations on gene expression. RT-qPCR was used to quantify mRNA levels of CCN1, Fibronectin, E-cadherin, and N-cadherin in BEAS-2B cells exposed to TGF-β1 at 0, 5, 10, and 20 ng/ml for 48 h. (**B**, **C**) Corresponding changes in protein expression were assessed by Western blot (**B**) and quantified by densitometry (**C**) under the same experimental conditions (0–20 ng/ml, 48 h). (**D**) Temporal dynamics of gene expression. BEAS-2B cells were stimulated with 10 ng/ml TGF-β1, and RT-qPCR was performed at 6, 12, and 24 h to track gene expression kinetics. (**E**, **F**) The kinetics of protein expression were evaluated by Western blot (**E**) and densitometry (**F**) in cells exposed to 10 ng/ml TGF-β1 for 12, 24, and 48 h. For all blots, GAPDH served as the internal loading control. All quantitative data represent the mean ± SD of three independent experiments (*n* = 3). Statistical comparisons were made using one-way ANOVA with Tukey’s multiple comparisons test. ^*^P < 0.05, ^**^P < 0.01 vs. the untreated control group

To investigate the dynamics of CCN1 expression in response to TGF-β1, we stimulated BEAS-2B cells with varying concentrations of TGF-β1 (0, 5, 10, 20 ng/ml) for 24 h. QPCR analysis revealed a significant induction of CCN1 mRNA, which peaked at 5 ng/ml but notably declined at higher concentrations (20 ng/ml). This was distinct from the expression of Fibronectin, which showed a sustained increase up to 20 ng/ml (Fig. 5A). Consistent with the transcriptional data, Western blot analysis demonstrated a differential sensitivity profile: while CCN1 protein levels showed maximal induction at 5–10 ng/ml and decreased at the highest concentration (20 ng/ml), the structural markers Fibronectin and N-cadherin exhibited a sustained elevation that persisted at 20 ng/ml, concomitant with the dose-dependent suppression of E-cadherin (Fig. 5B and C).

Next, we examined the temporal regulation of CCN1. BEAS-2B cells were treated with 10 ng/ml TGF-β1 for different durations. Consistent with the kinetics of immediate-early genes, the induction of CCN1 mRNA was rapid, peaking as early as 6 h post-stimulation, whereas Fibronectin mRNA accumulated progressively over the 48-hour period (Fig. 5D). Western blot analysis confirmed the subsequent protein translation kinetics: consistent with the temporal lag between transcription and translation, CCN1 protein accumulation followed the mRNA peak, becoming prominent at 24 h and sustained through 48 h, while Fibronectin and N-cadherin protein levels increased steadily throughout the time course (Fig. 5E and F).

### CCN1 is a critical mediator of the TGF-β1-induced pro-fibrotic program

Having established that TGF-β1 induces CCN1, we next asked whether CCN1 is a functionally essential mediator of the pro-remodeling effects of TGF-β1. To address this, we used both loss- and gain-of-function approaches in BEAS-2B cells.

**Fig. 6:**
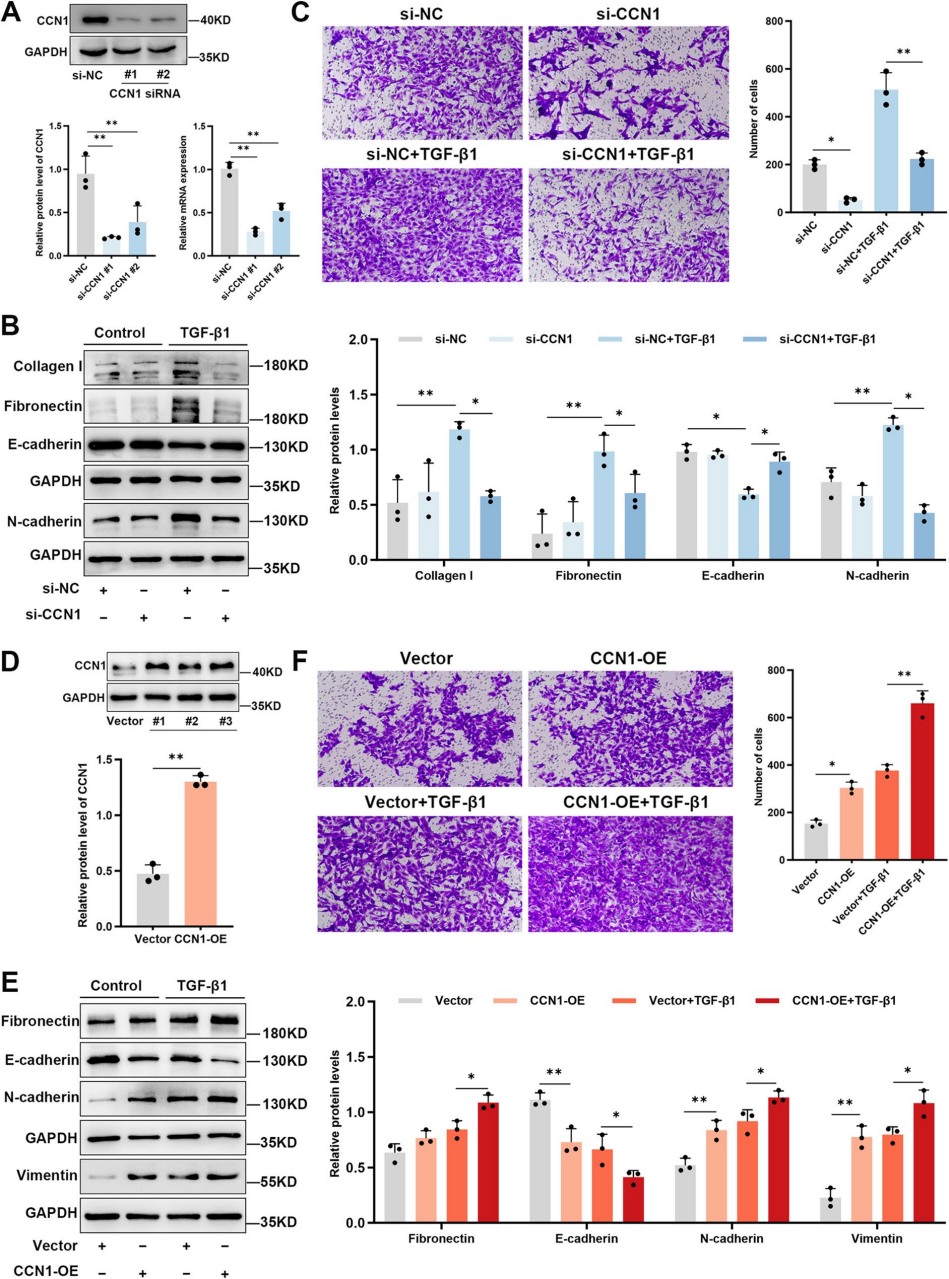
CCN1 is an essential mediator of TGF-β1-driven EMT and migration in human bronchial epithelial cells. (**A**–**C**) Loss-of-function studies demonstrating the requirement of CCN1 for TGF-β1-mediated effects. (**A**) Verification of CCN1 knockdown efficiency by Western blot (top) and RT-qPCR (bottom) in BEAS-2B cells transfected with a non-targeting si-NC or two distinct CCN1-targeting siRNAs (#1, #2). (**B**) Immunoblot analysis and corresponding quantification showing that CCN1 silencing attenuates the TGF-β1-induced changes in Collagen I, Fibronectin, E-cadherin, and N-cadherin expression. Cells were transfected as indicated and then stimulated with 10 ng/ml TGF-β1 for 48 h. (**C**) Representative micrographs and quantification of a Transwell migration assay, revealing that CCN1 knockdown impairs the pro-migratory effect of TGF-β1 (**D**–**F**) Complementary gain-of-function studies confirming the sufficiency of CCN1 to drive pro-remodeling phenotypes. (**D**) Validation of CCN1 overexpression (OE) via Western blot compared to an empty vector control. (**E**) Immunoblot analysis and quantification demonstrating that CCN1 overexpression, even in the absence of TGF-β1, is sufficient to induce changes in Fibronectin, E-cadherin, N-cadherin, and Vimentin. Cells were transfected with either an empty vector or a CCN1 OE plasmid before stimulation with or without 10 ng/ml TGF-β1 for 48 h. (**F**) A Transwell migration assay showing that CCN1 overexpression enhances cellular migration independently and additively with TGF-β1. For all immunoblots, GAPDH was used as the internal loading control. All quantitative results are expressed as mean ± SD of three independent experiments (*n* = 3), with individual data points shown as dots. Group comparisons were made using one-way ANOVA followed by Tukey’s multiple comparisons test (or unpaired t-test for Panel D). ^*^P < 0.05 and ^**^P < 0.01 were considered statistically significant vs. the relevant control

First, we silenced CCN1 using siRNAs and confirmed high knockdown efficiency (Fig. 6A). As hypothesized, CCN1 knockdown significantly blunted the pro-fibrotic and EMT-like effects of TGF-β1. While TGF-β1 robustly suppressed E-cadherin and upregulated N-cadherin, Fibronectin, and Collagen I in control cells, these changes were markedly attenuated upon CCN1 silencing (Fig. 6B). Consistent with this molecular reversal, a Transwell assay showed that CCN1 knockdown significantly impaired not only the TGF-β1-driven migration but also the basal cell motility (Fig. 6C). These results indicate that CCN1 is necessary for the full execution of the TGF-β1-driven pro-remodeling program and contributes to intrinsic epithelial cell motility.

Conversely, we tested whether CCN1 is sufficient to drive these changes by overexpressing it in BEAS-2B cells (Fig. 6D). Indeed, CCN1 overexpression alone was sufficient to induce an EMT-like state, characterized by decreased E-cadherin and increased N-cadherin, Fibronectin and Vimentin (Fig. 6E). Moreover, CCN1 overexpression potentiated the effects of TGF-β1, leading to a more pronounced EMT phenotype and a greater induction of Fibronectin. Functionally, this was mirrored by a significant increase in cell migration, which was further augmented by co-treatment with TGF-β1 (Fig. 6F). Together, these complementary experiments establish CCN1 as both a necessary and sufficient mediator of the TGF-β1-driven pro-fibrotic program and migratory phenotype in human bronchial epithelial cells.

### CCN1 amplifies TGF-β1 signaling via a Smad3-mediated positive feedback loop

Our finding that CCN1 acts as a crucial mediator of the TGF-β1 response led us to investigate whether it functions solely as a terminal effector or if it could also actively amplify the upstream signal. Given the canonical role of Smad3 phosphorylation in TGF-β1 signaling, we therefore hypothesized that a positive feedback loop might exist, whereby TGF-β1-induced CCN1 could in turn enhance Smad3 activation.

**Fig. 7:**
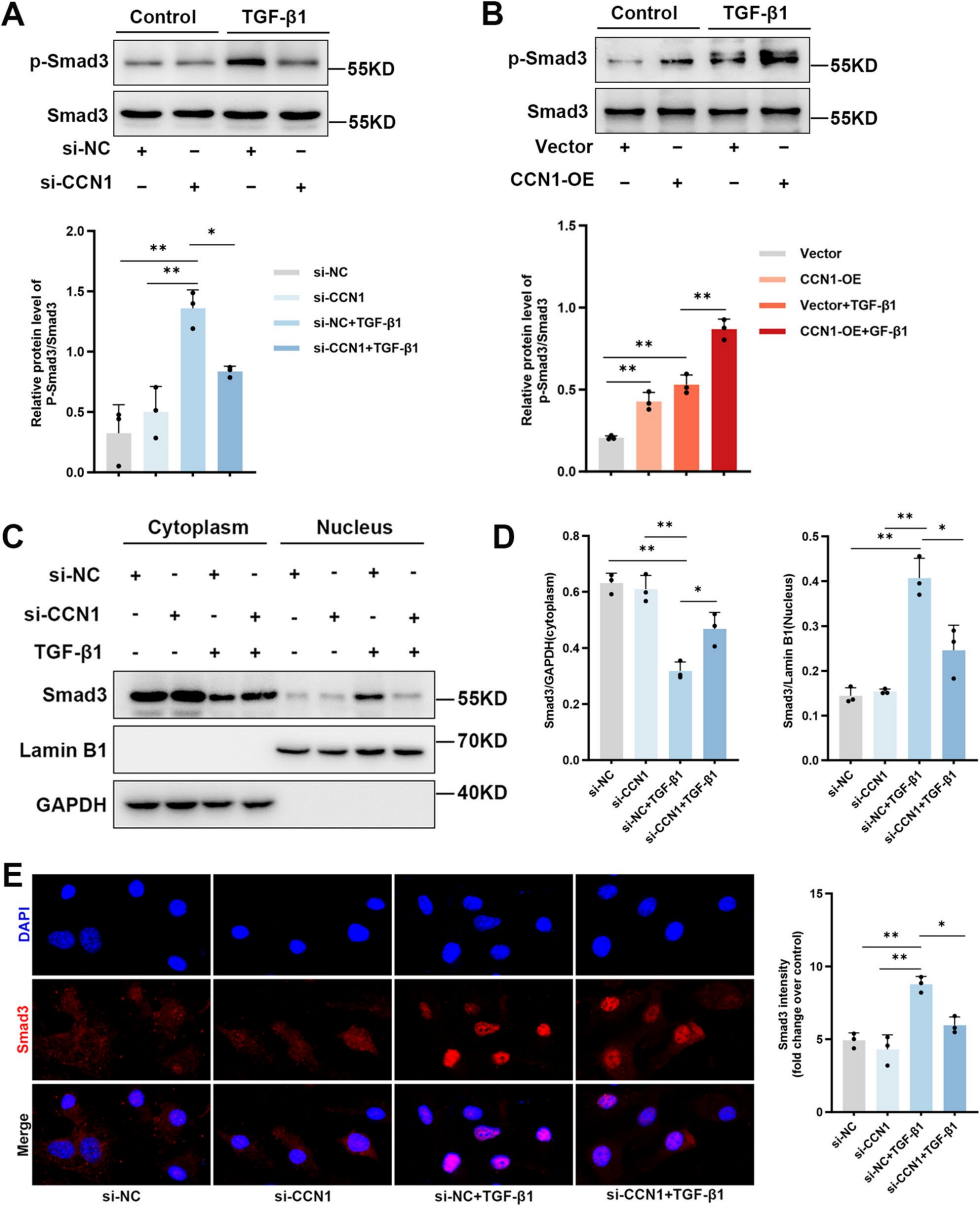
CCN1 amplifies TGF-β1 signaling by promoting Smad3 phosphorylation and nuclear translocation. (**A**) Representative Western blot and corresponding densitometric analysis of phosphorylated Smad3 (p-Smad3) levels in BEAS-2B cells transfected with either si-NC or si-CCN1, followed by stimulation with or without 10 ng/ml TGF-β1. Total Smad3 served as the loading control. (**B**) Parallel analysis assessing p-Smad3 levels in cells transfected with either a control vector or a CCN1 OE plasmid, following stimulation with or without TGF-β1. (**C**, **D**) Subcellular localization of Smad3 was assessed by nuclear/cytoplasmic fractionation. Representative immunoblots (**C**) and quantification of Smad3 levels in the cytoplasmic and nuclear fractions (**D**) are shown. The purity of the fractions was validated using GAPDH (cytoplasmic marker) and Lamin B1 (nuclear marker). (**E**) Immunofluorescence microscopy was used to visualize Smad3 nuclear translocation. Representative images (left) and quantification of nuclear fluorescence intensity (right) are displayed for cells treated as indicated. Cells were stained for Smad3 (red), and nuclei were visualized with a DAPI counterstain (blue). Scale bar = 50 μm. All quantitative data are expressed as mean ± SD from three independent experiments (*n* = 3). Statistical comparisons were performed using one-way ANOVA with Tukey’s multiple comparisons test. ^*^P < 0.05; ^**^P < 0.01 vs. the relevant control group

To test this, we first examined the effect of CCN1 knockdown on Smad3 phosphorylation. As expected, TGF-β1 induced a robust phosphorylation of Smad3 in control cells. This response, however, was significantly blunted in cells where CCN1 was silenced (Fig. 7A). Conversely, CCN1 overexpression alone was sufficient to increase basal p-Smad3 levels and, when combined with TGF-β1, markedly amplified the phosphorylation signal compared to control cells (Fig. 7B). Notably, these in vitro findings were recapitulated in our mouse models (see Additional file 1: Figure S2 A-D).

The critical step following Smad3 phosphorylation is its translocation into the nucleus. We therefore assessed if CCN1 influenced this process. Using nuclear/cytoplasmic fractionation, we observed that TGF-β1-induced nuclear accumulation of Smad3 was markedly inhibited by CCN1 silencing (Fig. 7C and D). Immunofluorescence staining visually confirmed this finding, showing that the prominent nuclear Smad3 signal seen upon TGF-β1 stimulation was substantially diminished in cells lacking CCN1 (Fig. 7E). These results unveil a novel positive feedback mechanism: CCN1, itself a TGF-β1 target, acts to amplify the upstream signal by promoting the phosphorylation and subsequent nuclear translocation of Smad3, thereby creating a self-sustaining pro-fibrotic loop.

## Discussion

This study identifies the matricellular protein CCN1 as a novel and critical driver of airway remodeling in asthma. Our data demonstrate a significant upregulation of CCN1 in the airways of both human asthmatics and a corresponding murine model. Mechanistically, we show that elevated CCN1 levels promote both EMT and the deposition of ECM, which are hallmark features of airway remodeling [19]. Central to our findings is a dual mode of action: CCN1 possesses intrinsic capacity to drive EMT-like changes independently, while simultaneously functioning within a positive feedback loop to amplify the canonical TGF-β1/Smad3 signaling cascade [20, 21].

Our in vivo work strongly supports a pro-remodeling role for CCN1. In our OVA-challenged mouse model, the upregulation of CCN1 in the airway epithelium directly correlated with increased levels of fibrotic and mesenchymal markers, including Fibronectin, Collagen I, and α-SMA. To test whether this link was causal, we performed direct interventions. Indeed, knocking down CCN1 with a lentivirus significantly blunted airway remodeling. Conversely, administering recombinant CCN1 protein intranasally significantly exacerbated the pathological changes induced by allergic inflammation. Together, these gain- and loss-of-function experiments pinpoint CCN1 as a key driver of the structural damage seen in asthmatic airways.The capacity of CCN1 to promote the deposition of key ECM components aligns with its established role as a pro-fibrotic mediator in other chronic diseases, including idiopathic pulmonary fibrosis (IPF) [22], liver cirrhosis, and renal fibrosis [23, 24].

Here, we extend this known pro-fibrotic function of CCN1 to the context of allergic airway disease. Central to the pro-remodeling function of CCN1 is its ability to drive airway remodeling primarily by inducing EMT in airway epithelial cells. Crucially, our data reveal that CCN1 is not merely a passive downstream effector. Across both in vitro and in vivo settings, CCN1 orchestrates a canonical EMT program characterized by the loss of E-cadherin and the concomitant gain of N-cadherin and Vimentin. Notably, our in vitro results demonstrated that silencing CCN1 suppressed basal cell migration even in the absence of TGF-β1, whereas CCN1 overexpression alone was sufficient to trigger mesenchymal marker expression. This indicates that CCN1 exhibits intrinsic biological effects independent of exogenous TGF-β1 stimulation [25, 26]. While this pro-EMT function of CCN1 is well-established in cancer metastasis [26], our work provides the first direct evidence that this same mechanism—acting both independently and synergistically—is a critical driver of pathogenesis in asthma.

Perhaps the most significant contribution of our work is the elucidation of the synergy between CCN1 and the canonical TGF-β1/Smad3 signaling pathway, a central axis in fibrosis and airway remodeling [27, 28]. The relationship between CCN1 and TGF-β1 has been described as complex and context-dependent, with some reports even suggesting an antagonistic interaction [29–31]. Our study brings clarity to this relationship in airway epithelial cells. Contrary to a simple linear dose-dependency, we observed that TGF-β1 induces CCN1 expression with distinct kinetics and threshold effects, peaking at specific physiological concentrations (5 ng/ml) to act as an early “trigger”. This pattern is consistent with the regulatory dynamics of immediate-early genes (IEGs), which often engage negative feedback loops to limit excessive signaling. In contrast, the sustained elevation of Fibronectin at high TGF-β1 concentrations likely reflects the cumulative deposition of stable ECM components, which persists even when the immediate-early response (CCN1) is attenuated. Once expressed, CCN1 then feeds back to potentiate the pathway by promoting the phosphorylation and nuclear import of Smad3. This circuit, where CCN1 both executes (via independent EMT induction) and reinforces (via Smad3 amplification) TGF-β1 signals, offers a compelling explanation for the persistent and progressive nature of airway remodeling in chronic asthma [32]. By effectively “locking in” a pro-fibrotic state, the CCN1-TGF-β1 axis emerges as a high-value target for therapeutic intervention.

While our study reveals this feedback loop, the precise mechanism by which extracellular CCN1 enhances Smad3 activation warrants further investigation. As a matricellular protein, CCN1 exerts its functions primarily by binding to cell surface integrin receptors [14, 33]. We speculate that CCN1 may engage specific integrin receptors on bronchial epithelial cells, triggering intracellular signaling cascades that cross-talk with the TGF-β receptor complex. This interaction could, for instance, involve kinases such as FAK or Src, which have been previously implicated in integrin and TGF-β signaling crosstalk [34, 35], thereby potentiating Smad3 phosphorylation. Delineating this specific receptor-mediated pathway will be an important direction for future studies.

Certain limitations of our study should be considered as they highlight important avenues for future research. First, regarding our in vivo gain-of-function experiments, we focused on the exacerbation of OVA-induced remodeling by recombinant CCN1. While our in vitro data confirmed the sufficiency of CCN1 to induce EMT-like changes, a “rmCCN1 alone” group in the animal model would have further delineated its sufficiency in vivo, representing a limitation to be addressed in follow-up studies. Second, our investigation focused on the canonical Smad pathway; however, given that TGF-β1 also activates non-Smad pathways [36, 37], exploring whether CCN1 modulates these cascades is a logical next step. Furthermore, this study centered on the structural impact of CCN1 on the epithelium, but its potential interplay with the broader inflammatory milieu, including effects on immune cell recruitment and function, remains to be determined. Finally, while our initial findings in human patient samples are compelling, validation in larger, well-phenotyped patient cohorts is essential to fully establish the clinical relevance of the CCN1-TGF-β1 axis and its potential as a biomarker.

## Conclusions

Taken together, our findings establish CCN1 as a central player in asthmatic airway remodeling. We have elucidated a molecular mechanism in which CCN1 and TGF-β1 form a positive feedback loop that drives a persistent pro-fibrotic state. This work provides a strong rationale for developing novel therapies aimed at disrupting this axis to treat progressive airway obstruction in asthma [38].

## Supplementary Information

Additional file 1: Table S1. A comprehensive summary of the clinical characteristics of all human study participants. Table S2. siRNA sequences for gene silencing. Table S3. Primers for qRT-PCR. Figure S1. Validation of Lentiviral-Mediated CCN1 Knockdown Efficacy in vivo. Figure S2. Effect of CCN1 modulation on Smad3 phosphorylationin vivo. Figure S1.Validation of Lentiviral-Mediated CCN1 Knockdown Efficacyin vivo. (A, B) To confirm successful target gene suppression, we performed immunofluorescence microscopy on lung sections. These representative images and the associated quantification demonstrate that therapeutic delivery of LV2-shCCN1 resulted in a significant reduction of CCN1 protein expression specifically within the bronchial epithelium of OVA-challenged mice when compared to the non-targeting control. Scale bar = 50 µm. All quantitative data are expressed as the mean ± SD from three independent animal cohorts. Statistical significance was assessed using one-way ANOVA followed by Tukey's multiple comparisons test. ^*^P < 0.05; ^**^P< 0.01 versus the shNC-treated group. Figure S2. Effect of CCN1 modulation on Smad3 phosphorylation in vivo. (A, B) Immunoblotting of whole-lung homogenates revealed that lentiviral-mediated knockdown of CCN1 significantly attenuated the OVA-induced phosphorylation of Smad3 relative to the non-targeting control. (C, D) Conversely, gain-of-function experiments involving the intranasal administration of rmCCN1 produced a marked increase in Smad3 phosphorylation over that observed in the OVA-challenged group that received vehicle alone. All graphical data represent the mean ± SD from three separate experiments. Group-wise statistical comparisons were performed using one-way ANOVA followed by Tukey's multiple comparisons test. ^*^P < 0.05 and ^**^P < 0.01 were considered statistically significant versus the relevant control group.

## Data Availability

All data generated or analysed during this study are included in this published article [and its supplementary information files].

## Abbreviations

ECM: extracellular matrix
CCN1: Cellular Communication Network Factor 1
EMT: epithelial-mesenchymal transition
TGF-β1: transforming growth factor-β1
OVA: ovalbumin
AHR: airway hyperresponsiveness
siRNA: small interfering RNA
CCN1 OE: CCN1 overexpression
rmCCN1: Recombinant mouse CCN1
shRNA: short hairpin RNA
IHC: Immunohistochemistry
IF: Immunofluorescence
