# Supplementary material for: CCN1 drives asthmatic airway remodeling through amplification of TGF-β1/Smad3 signaling
Source: Respir Res. 2026 Jan 20;27:62. doi: 10.1186/s12931-026-03506-8 (PMC12895598; doi:10.1186/s12931-026-03506-8)
Supplement: Supplementary file 1 — Additional file 1: Table S1. A comprehensive summary of the clinical characteristics of all human study participants. Table S2. siRNA sequences for gene silencing. Table S3. Primers for qRT-PCR. Figure S1. Validation of Lentiviral-Mediated CCN1 Knockdown Efficacy in vivo. Figure S2. Effect of CCN1 modulation on Smad3 phosphorylationin vivo. Figure S1.Validation of Lentiviral-Mediated CCN1 Knockdown Efficacyin vivo. (A, B) To confirm successful target gene suppression, we performed immunofluorescence microscopy on lung sections. These representative images and the associated quantification demonstrate that therapeutic delivery of LV2-shCCN1 resulted in a significant reduction of CCN1 protein expression specifically within the bronchial epithelium of OVA-challenged mice when compared to the non-targeting control. Scale bar = 50 µm. All quantitative data are expressed as the mean ± SD from three independent animal cohorts. Statistical significance was assessed using one-way ANOVA followed by Tukey's multiple comparisons test. *P < 0.05; **P< 0.01 versus the shNC-treated group. Figure S2. Effect of CCN1 modulation on Smad3 phosphorylation in vivo. (A, B) Immunoblotting of whole-lung homogenates revealed that lentiviral-mediated knockdown of CCN1 significantly attenuated the OVA-induced phosphorylation of Smad3 relative to the non-targeting control. (C, D) Conversely, gain-of-function experiments involving the intranasal administration of rmCCN1 produced a marked increase in Smad3 phosphorylation over that observed in the OVA-challenged group that received vehicle alone. All graphical data represent the mean ± SD from three separate experiments. Group-wise statistical comparisons were performed using one-way ANOVA followed by Tukey's multiple comparisons test. *P < 0.05 and **P < 0.01 were considered statistically significant versus the relevant control group. [file 12931_2026_3506_MOESM1_ESM.zip › Additional file 1/Additional file 1 Fig S1.pdf]

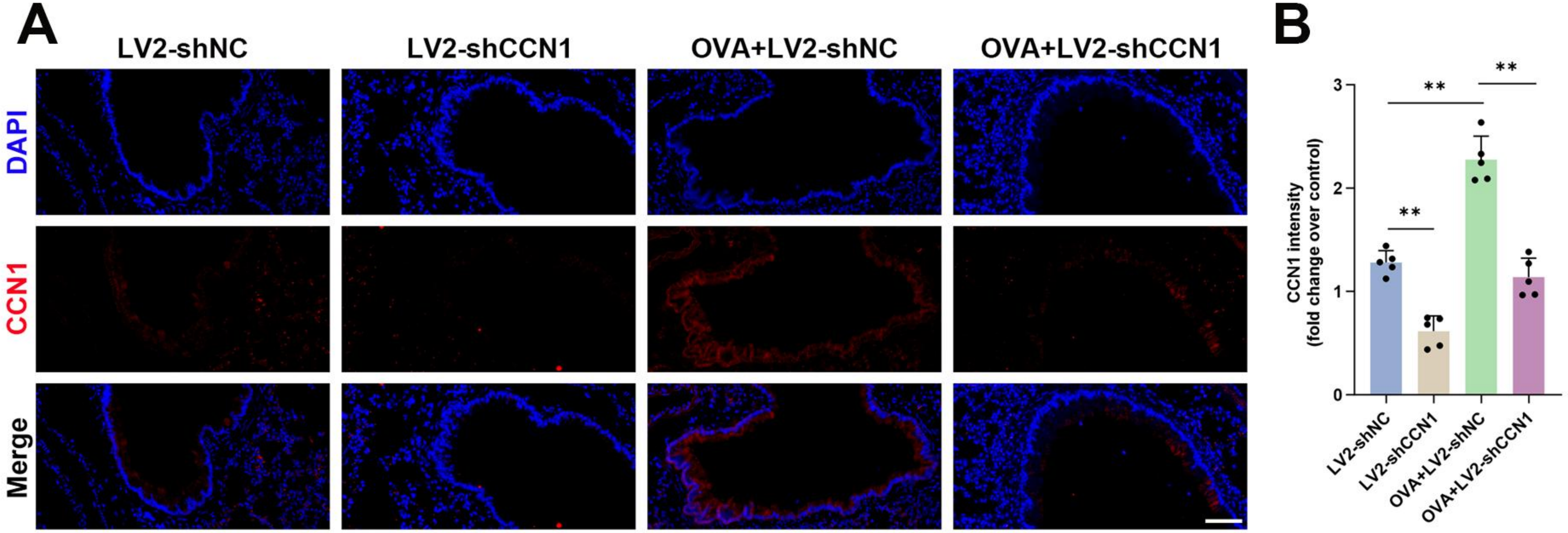

Figure S1.Validation of Lentiviral-Mediated CCN1 Knockdown Efficacy *in vivo*.
(A, B) To confirm successful target gene suppression, we performed immunofluorescence microscopy on lung sections. These representative images and the associated quantification demonstrate that therapeutic delivery of LV2-shCCN1 resulted in a significant reduction of CCN1 protein expression specifically within the bronchial epithelium of OVA-challenged mice when compared to the non-targeting control. Scale bar = 50 μm. All quantitative data are expressed as the mean ± SD from three independent animal cohorts. Statistical significance was assessed using one-way ANOVA followed by Tukey's multiple comparisons test. *P < 0.05; **P < 0.01 versus the shNC-treated group.
